# Melatonin Modulates the SIRT1-Related Pathways via Transdermal Cryopass-Laser Administration in Prostate Tumor Xenograft

**DOI:** 10.3390/cancers15204908

**Published:** 2023-10-10

**Authors:** Francesca Bonomini, Gaia Favero, Anna Petroni, Rita Paroni, Rita Rezzani

**Affiliations:** 1 Anatomy and Physiopathology Division, Department of Clinical and Experimental Sciences, University of Brescia, 25123 Brescia, Italy; francesca.bonomini@unibs.it (F.B.); gaia.favero@unibs.it (G.F.); 2 Interdipartimental University Center of Research “Adaption and Regeneration of Tissues and Organs (ARTO)”, University of Brescia, 25123 Brescia, Italy; 3 Italian Society for the Study of Orofacial Pain (Società Italiana Studio Dolore Orofacciale—SISDO), 25123 Brescia, Italy; 4 Biomedicine and Nutrition Research Network, Via Paracelso 1, 20129 Milan, Italy; annapetroni@gmail.com; 5 Department of Medicine, Surgery and Neuroscience, University of Siena, 53100 Siena, Italy; 6 Clinical Biochemistry and Mass Spectrometry, Department of Health Sciences, San Paolo Hospital, Università degli Studi di Milano, 20142 Milan, Italy; rita.paroni@unimi.it

**Keywords:** melatonin, oxidative stress, tumor, sirtuin1, proliferator-activated receptor gamma, PPAR-gamma coactivator 1-alpha, nuclear factor kappa B

## Abstract

**Simple Summary:**

The likelihood of prostate tumor diagnosis in men increases with age, raising the need for novel therapies to better treat this disease. This research examined mechanisms on the basis of the anti-tumor effects of melatonin against prostate tumor cells xenografted into nude mice when delivered by a new and alternative route based on transdermal administration by cryopass-laser treatment. Despite other signaling pathways targeted by this molecule that have already been described, here we demonstrate that the significant anti-proliferative effect displayed by transdermal melatonin is also due to the Sirtuin1 pathway modulation.

**Abstract:**

Melatonin displays antitumor activity in several types of malignancies; however, the best delivery route and the underlying mechanisms are still unclear. Alternative non-invasive delivery route based on transdermal administration of melatonin by cryopass-laser treatment demonstrated efficiency in reducing the progression of LNCaP prostate tumor cells xenografted into nude mice by impairing the biochemical pathways affecting redox balance. Here, we investigated the impact of transdermal melatonin on the tumor dimension, microenvironment structure, and SIRT1-modulated pathways. Two groups (vehicle cryopass-laser and melatonin cryopass-laser) were treated for 6 weeks (3 treatments per week), and the tumors collected were analyzed for hematoxylin eosin staining, sirius red, and SIRT1 modulated proteins such as PGC-1α, PPARγ, and NFkB. Melatonin in addition to simple laser treatment was able to boost the antitumor cancer activity impairing the tumor microenvironment, increasing the collagen structure around the tumor, and modulating the altered SIRT1 pathways. Transdermal application is effective, safe, and feasible in humans as well, and the significance of these findings necessitates further studies on the antitumor mechanisms exerted by melatonin.

## 1. Introduction

Prostate tumor is the most diagnosed male malignancy and ranks second in global tumor incidence [1,2]. Old age, family history, obesity, hypertension, lack of exercise, chronically high testosterone levels, and ethnicity are the main known prostate tumor risk factors [3]. Fortunately, while the incidence of tumors rises with age, the aggressiveness of the disease is generally lower in elderly patients [4].

Prostate tumor is thought to be caused by a variety of complex mechanisms that include numerous cell signaling pathways, according to in vitro and in vivo research [5].

Many signaling pathway elements represent viable therapeutical targets, and are present within the tumor cell, on its surface as receptors, or in the environment as ligands. These targets affect proliferation, differentiation, and cell death. These targets involve angiogenesis, immune function, and tumor invasion. Thus, most targets are directly or indirectly critical to some aspect of tumor cell physiology [6]. 

Melatonin is a pleiotropic and multitasking indoleamine that, apart from circadian regulations, has antioxidant, anti-aging, and antitumor properties [7,8]. Even if a disruption in pineal melatonin production and circadian rhythmicity has been shown to increase tumor risk, the antitumor melatonin mechanism(s) of action are not yet fully known [9]. Recently, a new and alternative melatonin delivery route based on transdermal administration by cryopass-laser treatment has shown promising effects in reducing the progression of tumors; however, the underlying pathways involved are not yet completely characterized [10].

Among the proteins previously described to be involved in tumor development and progression are Sirtuins (SIRTs), a group of proteins involved in different pathologies such as metabolic disorders and tumors. Among the seven SIRTs (classified from SIRT1 to SIRT7), SIRT1 was well-studied [11]. SIRT1 interacts with protein substrates in a variety of signaling pathways and plays a central regulatory role in cell proliferation, differentiation, senescence, inflammation, and apoptosis.

Interestingly, SIRT1 expression was known to be significantly increased in many tumors, such as colon tumors, leukemia, non-melanoma skin cancer, and prostate tumors [12,13].

Moreover, SIRT1 can deacetylate some important transcriptional factors in both metabolism-dependent and independent metabolic pathways, such as peroxisome proliferator-activated receptor gamma (PPAR-γ) and PPAR-gamma coactivator 1-alpha (PGC-1α), to affect mitochondrial function and biogenesis [14].

SIRT1 can also modulate Nuclear Factor kB (NF-kB), which is considered the central mediator of the inflammatory process and innate immunity, contributing to important cellular processes. NF-kB, a p65/p50 heterodimer, is usually retained in the cytoplasm bound to the NF-kB inhibitor (IkB). The p65 subunit is targeted by SIRT1, which, through deacetylation, unmasks the nuclear localization sequence of NF-κB, thereby allowing it to enter the nucleus and regulate the transcription level of some inflammatory and antiapoptotic genes [15].

Furthermore, recent evidence has suggested that SIRT1 regulates expression of the metalloproteinase 2 (MMP2), also termed Gelatinase A, a zinc-dependent endopeptidase that is involved in the degradation of various proteins in the extracellular matrix (ECM) [16]. 

MMP2-mediated proteolytic remodeling of the ECM and changes in collagen content provide a mechanism for regulating directional cell motility and tumor invasion in prostate cancer [16,17].

In this in vivo study, we evaluated the melatonin antitumor activity using an alternative noninvasive delivery route against prostate tumor cells (LNCaP) xenografted into nude mice and, subsequently, we tried to shed light on the melatonin mechanism(s) of action by looking at the posttranslational regulation of SIRT1.

For this aim, we used Foxn1^nu/nu^ mice, which represents a widely used model in cancer research that has a long-lasting history as a tool for in vivo testing of anticancer therapies [18].

Here, we report that melatonin delivered by cryopass laser acts as a novel SIRT1 inhibitor and imparts anti-proliferative effects, as demonstrated by the decrease in the nuclear marker for cell proliferation Ki67, in prostate tumor xenografted in mice via inhibiting SIRT1, which in turn acts on ECM, PGC-1α/PPAR-γ pathway and modulates NF-κB signaling.

## 2. Materials and Methods

### 2.1. Experimental Groups

Twenty male Foxn1^nu/nu^ mice of seven-week of age (Harlan Laboratories, Indianapolis, IN, USA) were housed in standard plastic cages (five animals/cage) in a temperature-controlled animal facility with a 12 h alternating light–dark cycle to minimize circadian variations. As previously reported by Paroni et al. [8] and Terraneo et al. [10], water and bedding were heat-sterilized, and food was sterilized by 60Co γ-irradiation. 

The experimental animals have been cared for following the Guide for the Care and Use of Laboratory Animals published by the National Institutes of Health (NIH Publication No. 85-23, revised 1996). The Ethical Committee of the University of Milan (Italy) approved the experimental protocol (All.5 verb.16.03.2010), and all efforts were made to minimize animal suffering and the number of animals used.

This animal strain presents nude mice, which are hairless from birth throughout life and show an underdeveloped thymus. Given the absence of Foxn1 protein due to nu/nu recessive mutation, these animals have a T lymphocyte deficiency, and the reduced lymphocyte population is composed almost entirely of B-cells. 

This model has certain limitations because the immunodeficiency is severe, but not absolute. Even though there are only a few T cells in the periphery, the intact innate immunity, particularly high NK cell activity, can limit the engraftment take rate. The natural immunosuppression of mice allows for a relatively simple-to-achieve engraftment of a desired type of tumor by simply inoculating the cancer cells into the animal host. Moreover, due to the natural lack of hair, the tumor growth can be easily monitored in athymic nude mice when injected subcutaneously [18]. 

**Figure 1:**
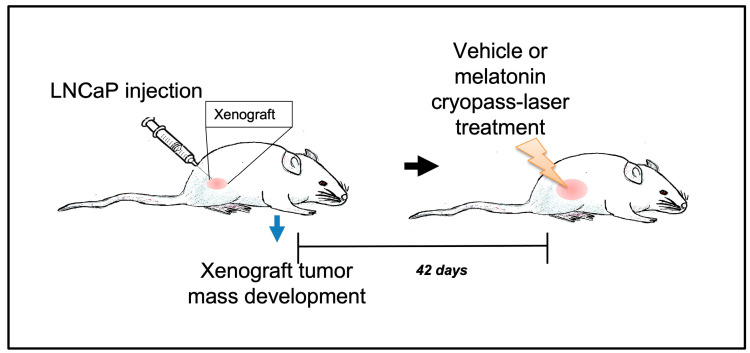
Animal treatment scheme.

All mice received bilateral LNCaP cells inoculation (3 × 10^6^ cells in 0.1 mL) in the flank (the detailed procedure of LNCaP cells culture was previously described by Paroni et al. [8] and Terraneo et al. [10]), and then the experimental animals were randomly divided into the vehicle cryopass-laser-treated group (*n* = 10 animals) or melatonin cryopass-laser-treated group (*n* = 10 animals) (Figure 1). The equipment for cryopass-laser treatment (LASERICE Med C.I.R.C.E. S.r.L., Magnago, Milano, Italy) was constituted by cryo applicators and by a scanner connected to a photodiode laser beam generator with λ 635 nm, maximum power < 5 mW, collimation lens < 20 mV. Before the beginning of the treatment experiments, a suspension of 1.5% (*w/v*) hydroxymethyl cellulose containing melatonin (melatonin cryopass-laser-treated mice) or devoid of melatonin (vehicle cryopass-laser-treated mice) was prepared by emulsifying for 7 min in ice and dark with a disperser tool (Ultra-Turrax T25, IKA, Labortechnik, Staufen, Germany) at the maximum speed. Then, 15 mL of this suspension was transferred into cryo applicators and immediately frozen at −20 °C until use. The melatonin dose was 0.120 mg/mouse/each cryopass-laser treatment, corresponding to 4 mg/kg, i.e., a total amount of ~2.2 mg/mouse delivered at the end of the 18 treatments scheduled. All details on cryopass-laser treatments can be found elsewhere [10].

During the treatment period, body weight and xenograft volume were monitored 3 times/week for 42 days. 

At the end of the treatment period, each mouse was deeply anesthetized with sodium thiopental (10 mg/100 g body weight) plus heparin (500 units) and euthanized by cervical dislocation. The xenografts were carefully removed, weighed, washed in phosphate-buffered saline (PBS), fixed in formalin solution for 24 h, and embedded in paraffin wax following standard procedure [8,19].

Serial sections (7 μm thick) of each xenograft sample were cut with a microtome and submitted for morphological and immunofluorescence/immunohistochemistry evaluations.

### 2.2. Xenograft Morphological Evaluation

Alternate xenograft sections were deparaffinized, rehydrated, and stained with hematoxylin-eosin (following standard protocol) and Sirius red stainings. For Sirius red staining, the xenograft sections were stained for 5 min in 1% acid phosphomolybdic aqueous solution and then stained for 3 min in 6% Sirius red in an aqueous solution [20]. To distinguish between type I and type III collagen within tissue sections, we applied a Sirius polarization method (Sirius red staining combined with polarized light detection). With this method, type I collagen is detected in red-orange-yellow (thick fibers) and type III collagen is displayed in green (thin fibers) [21].

For the morphological evaluation, ten non-overlapping fields with the same area were randomly selected for each experimental animal and observed with an optical light microscope (Olympus, Hamburg, Germany) at a final magnification of 200× by two blinded investigators [21].

### 2.3. Immunofluorescence and Immunohistochemistry Assay

Alternate xenograft sections were deparaffinized, rehydrated, and subjected to antigen retrieval in 0.01 M sodium citrate buffer (pH 6.0) in a microwave oven. The sections were then washed in PBS, incubated in 3% hydrogen peroxide for 10 min, and subjected to a pre-absorption solution of 1% bovine serum albumin in 0.05% Tween 20 for 1 h at room temperature [22]. The xenograft sections were incubated for 1 h at 37 °C and for 1 h at room temperature with the following primary antibodies: rabbit polyclonal antibody against anti-Ki67 (diluted 1:100; Abcam, Cambridge, UK); anti-CD31; goat polyclonal antibody against MMP2 (diluted 1:200; Santa Cruz Biotechnology, Dallas, TX, USA); rabbit polyclonal antibody against SIRT1 (diluted 1:250; Abcam, Cambridge, UK); rabbit polyclonal antibody against PGC-1α (diluted 1:400; Abcam, Cambridge, UK); rabbit polyclonal PPAR**γ** (diluted 1:500; Abcam, Cambridge, UK; and rabbit polyclonal against NF-kB (diluted 1:350; Abcam, Cambridge, UK). 

For the immunofluorescence analysis, after rinsing with PBS, the xenograft sections were labeled with specific 488 Alexa Fluor-conjugated secondary antibodies (diluted 1:200–green staining; Invitrogen, Paisley, UK). Finally, the sections were counter-stained with 4′,6-diamidino-2-phenylindole (DAPI–blue staining), mounted, and observed with fluorescent microscopy (Nikon, Düsseldorf, Germany) at a final magnification of 400× by two blinded investigators [23].

For the immunohistochemical analysis, after primary antibody incubation, the sections were sequentially incubated in anti-rabbit biotinylated immunoglobulin and avidin-biotin peroxidase complex. The reaction products were visualized using 0.05% 3,3′-diaminobenzidine tetrahydrochloride (DAB), as chromogen and 0.33% hydrogen peroxide as catalysts [20]. The sections were finally counterstained with hematoxylin for CD31, MMP2, and NF-kB and with light green for Ki67, and were mounted and observed with a light microscope (Olympus BX50 microscope, Hamburg, Germany) at a final magnification of 400× and/or 1000× by two blinded investigators.

Sections without primary antibodies and in the presence of isotype-matched IgG served as negative immunofluorescence/immunohistochemistry controls.

Both for immunofluorescence and immunohistochemistry assays, three random fields from a total of two non-consecutive sections per animal were analyzed. In detail, for ki67 immunohistochemistry, the number of positive cells per field was evaluated; whereas the immunostaining for each other primary antibody (both for immunofluorescence and immunohistochemistry assays) was calculated using an image analyzer (Image Pro Premier 9.1, Media Cybernetics, Rockville, MD, USA) and expressed as an arbitrary unit (AU). Two blinded investigators performed the analysis, and their evaluations were assumed correct if the values were not significantly different. If there was disagreement concerning the interpretation, the case was reconsidered to reach a unanimous agreement [20].

### 2.4. Statistical Analysis

Results were expressed as the mean ± standard error of the mean (SEM). Data for multiple variable comparisons were analyzed by one-way analysis of variance (ANOVA corrected Bonferroni test). *p* ≤ 0.05 was considered significant for all statistical analyses in this study.

## 3. Results

### 3.1. Prostate Tumor Xenograft

**Figure 2:**
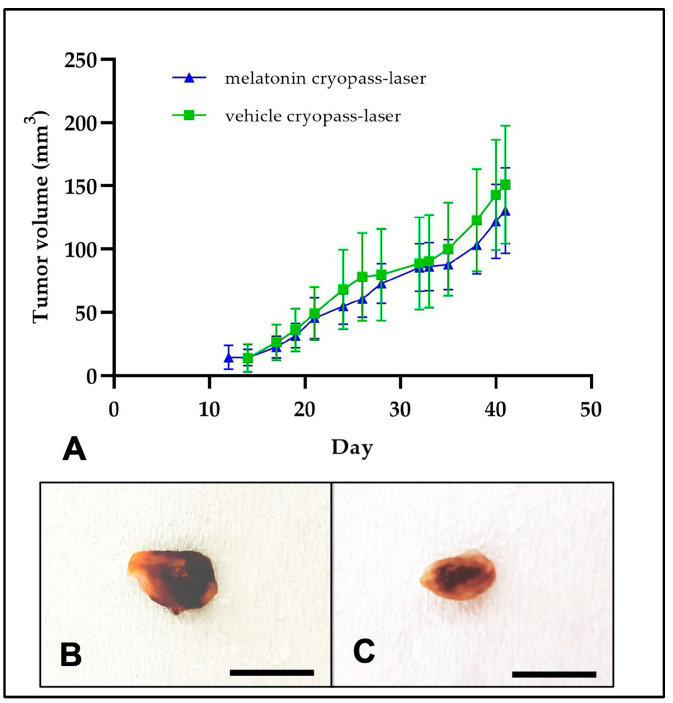
Graph of tumor growth (**A**). Representative images of excised tumors (after 42 days of treatment) from vehicle cryopass-laser-treated mice (**B**) and melatonin cryopass-laser-treated mice (**C**). Bars: 8 mm.

As previously reported, all mice survived treatments without evident signs of adverse effects, neither in observable changes in behavior, activity, or sign of stress, or infections such as skin redness or burns at the cryopass-laser site [8,10]. These findings confirmed, as previously reported by Terraneo et al. [10], that melatonin is well-tolerated at the dosage tested. Forty-two days of melatonin cryopass-laser treatment seem to counteract the body weight reduction induced by tumorigenesis, as observed also in our previous experiments when melatonin was delivered via i.p. administration [8]. Tumors became visible on the flank of cryopass-laser vehicle-treated mice group with the same kinetics observed on the cryopass-laser melatonin-treated mice. Melatonin cryopass-laser treatment inhibited the increase in tumor volume growth compared to vehicle cryopass-laser treatment (Table 1, Figure 2A). Figure 2B,C show representative images of excised tumors from vehicle cryopass-laser-treated mice (B) and melatonin cryopass-laser-treated mice (C).

Melatonin-specific tumor growth impairment was confirmed by morphological stainings and via Ki67 evaluation (known nuclear marker associated with cellular proliferation). 

**Figure 3:**
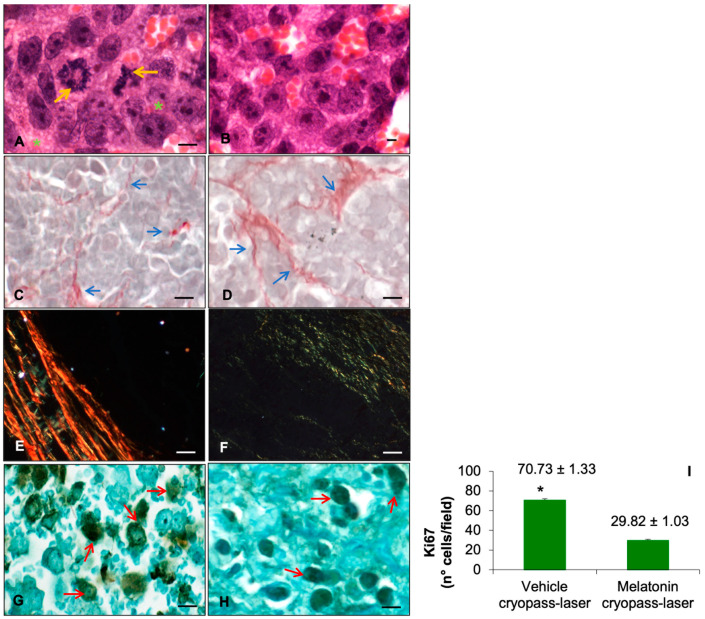
Representative xenograft photomicrographs of hematoxylin-eosin (**A**,**B**) and Sirius red (**C**,**D**), polarized Sirius red (**E**,**F**) morphological stainings and of Ki67 expression (**G**,**H**) on vehicle cryopass-laser-treated mice (**A**,**C**,**E**,**G**) and melatonin cryopass-laser-treated mice (**B**,**D**,**F**,**H**). Bars: 8 μm. The green asterisks indicate the extravascular red blood cells, the yellow arrows indicate the tumor cells nuclei with irregular shape, the blue arrows indicate collagen fibers, and the red arrows indicate Ki67 positivity. Graph (**I**) summarizes the number of Ki67 positive cells/field measurement. * *p* ≤ 0.05 vs. melatonin cryopass-laser group.

As reported in Figure 3, in both experimental groups, the xenograft tumor presented lobular organization as well as abundant extravascular red blood cells. Furthermore, tumor cells are characterized by a large nucleus with an irregular shape and size, exhibiting scarce cytoplasm and moderate neovascularization (Figure 3A). Instead, the xenografts in the group treated with melatonin cryopass-laser are characterized by nests of tumor cells and, notably, cryopass-laser melatonin treatment promotes tumor encapsulation (Figure 3B). In detail, compared to vehicle cryopass-laser tumors (Figure 3C), the cryopass-laser melatonin-treated xenografts appeared enclosed by collagen fibers (Figure 3D–visible in red by Sirius red staining).

The observation of collagen content using the Sirius polarization method showed the presence of type I collagen (Figure 3D–visible in red-orange-yellow) in prostate cancer tissue treated with vehicle cryopass-laser, while type III collagen (Figure 3E–visible in green) was present in melatonin cryopass-laser-treated tissue. Moreover, compact, crosslinked, and more oriented collagen was observed in the prostate cancer tissue treated with vehicle cryopass-laser compared to melatonin cryopass-laser-treated tissue.

Notably, the cellular proliferation, investigated via Ki67 expression, showed a significant presence in tumor cells of the vehicle cryopass-laser-treated group (Figure 3G); however, Ki67 positive cells displayed a significant decrease in the cryopass-laser melatonin-treated xenografts (Figure 3H). Graph 3I summarized the Ki67 quantification of Ki67 cell positivity and confirmed the observations reported.

**Figure 4:**
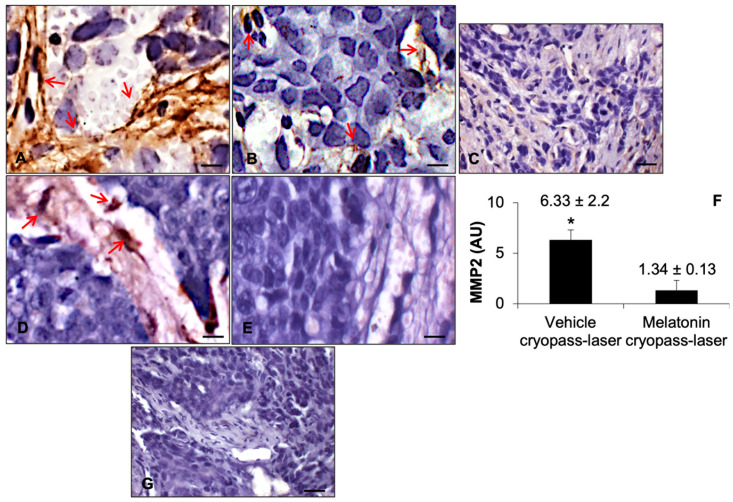
Representative photomicrographs of xenograft CD31 (**A**–**C**) and metalloproteinases2 (**D**,**E**,**G**) immunostainings of vehicle cryopass-laser-treated mice (**A**,**D**) and melatonin cryopass-laser-treated mice (**B**,**E**)–Bars: 8 μm. Representative photomicrographs of immunohistochemistry negative control-melatonin cryopass-laser-treated group (**C**) and vehicle cryopass-laser-treated group (**G**)—Bars: 20 μm. Graph (**F**) summarizes the metalloproteinase2 immunomorphometrical measurement. Red arrows indicate immunopositivity for CD31 (**A**,**B**) and MMP2 (**D**). * *p* ≤ 0.05 vs. melatonin cryopass-laser group. MMP2: metalloproteinase2.

To confirm the tumor neovascularization observed by the hematoxylin-eosin morphological staining, we also evaluated CD31 expression, a known marker of endothelial cells [24,25]. The vehicle cryopass-laser-treated group presented a significantly higher number of CD31-positive endothelial cells in the tumor (Figure 4A) compared to melatonin cryopass-laser-treated xenografts, which showed a very weak CD31 immunopositivity (Figure 4B). The CD31 expression confirmed the previous morphological observation of moderate neurovascolarization in the vehicle cryopass-laser-treated group. The negative controls of CD31 immunohistochemistry were similar in both vehicle and melatonin cryopass-laser-treated groups, and Figure 4C shows the melatonin cryopass-laser-treated xenograft.

Furthermore, to evaluate of the role of melatonin in the tumor embedding by collagen fibers observed in the melatonin cryopass-laser-treated by the Sirius red morphological staining in depth, we also evaluated the metalloproteinase2 (MMP2) expression (endopeptidases involved in collagen degradation) [26]. MMP2 was strongly expressed at the collagen encasement level of vehicle cryopass-laser-treated xenograft (Figure 4D). Interestingly, melatonin cryopass-laser-treated xenograft presented a very weak MMP2 immunopositivity (Figure 4E). These observations were confirmed by the immunomorphometrical analysis of xenograft MMP2 expression (Figure 4E). The above-reported observations were confirmed by the immunomorphometrical analysis of xenograft MMP2 expression (Figure 4F). The negative controls of MMP2 immunohistochemistry were similar in both vehicle and melatonin cryopass-laser-treated mice; Figure 4G shows the vehicle cryopass-laser-treated xenograft. 

### 3.2. Sirtuin1 in Tumorigenesis

**Figure 5:**
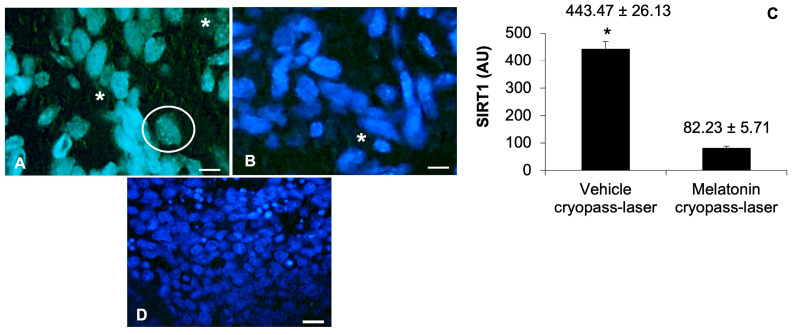
Representative photomicrographs of xenograft sirtuin1 immunostaining of vehicle cryopass-laser-treated mice (**A**) and melatonin cryopass-laser-treated mice (**B**)—Bars: 8 μm. Representative photomicrographs of immunofluorescence negative control-vehicle cryopass-laser-treated group (**D**). Bar: 20 μm. The white asterisks indicate the cytoplasmatic sirtuin1 positivity and the white circle indicates a representative sirtuin1 positive tumor cell nucleus. Graph (**C**) summarizes the sirtuin1 immunomorphometrical measurement. * *p* ≤ 0.05 vs. melatonin cryopass-laser group. SIRT1: sirtuin1.

To detect the underlying mechanism of SIRT1 in tumorigenesis, we investigated its expression using immunofluorescence assay. We observed a moderate/strong expression of SIRT1 at the xenograft level of vehicle cryopass-laser-treated mice (Figure 5A) compared to a very weak/absent expression at the xenograft level of melatonin cryopass-laser-treated mice (Figure 5B). In particular, at the xenograft level of the vehicle-treated group, SIRT1 immunopositivity was observed predominantly as moderate/strong nuclear export signals and weak as nuclear localization signals, indicative of SIRT1 translocation between the nucleus and cytoplasm, whereas the cryopass-laser melatonin-treated xenograft showed a very weak/absent SIRT1 immunopositivity mainly at cytoplasm level. The comparison among vehicle cryopass-laser and melatonin cryopass-laser-treated xenografts showed that SIRT1 immunopositivity is up-regulated in the vehicle-treated tumor cells with a significant difference compared to melatonin-treated tumors. The observations reported above were confirmed by the immunomorphometrical analysis of xenograft SIRT1 expression (Figure 5C). The negative controls of SIRT1 immunofluorescence were similar in both vehicle and melatonin cryopass-laser-treated mice; Figure 5D shows the vehicle cryopass-laser-treated xenograft.

### 3.3. Sirtuin1 Signaling Pathway in Tumorigenesis

**Figure 6:**
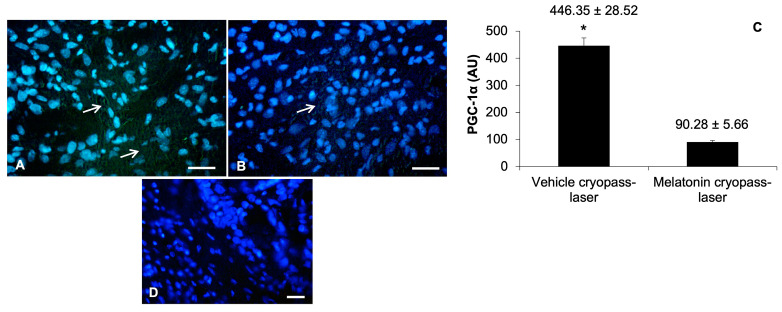
Representative photomicrographs of xenograft PGC-1α immunostainings of vehicle cryopass-laser-treated mice (**A**) and melatonin cryopass-laser-treated mice (**B**). Representative photomicrographs of the negative controls of PGC-1α immunofluorescence, (**D**)—vehicle cryopass-laser-treated group. Bars: 20 μm. The white arrows indicate the mitochondrial PGC-1α positivity. The graph (**C**) summarizes PGC-1α immunomorphometrical measurements. * *p* ≤ 0.05 vs. melatonin cryopass-laser group. PGC-1α: PPAR-gamma coactivator 1-alpha.

To confirm and investigate in depth the SIRT1-dependent PGC-1α activation and subsequent involvement of downstream proteins, we also analyzed PGC-1α and PPARγ expression by immunofluorescence and immunohistochemistry evaluations, respectively. Vehicle cryopass-laser-treated mice showed a moderate expression of PGC-1α at the tumor xenografted cell level (Figure 6A). Differently, tumors of the melatonin-treated mice presented a very weak/absent expression of PGC-1α (Figure 6B). In particular, the PGC-1α expression, which is a mitochondrial marker, was evident at the cytoplasm level of xenograft cells as “small spots” underlining its cytoplasmic organelle localization [19,20]. The observations reported above were confirmed by the immunomorphometrical analysis of xenograft PGC-1α expression (Figure 6C). The negative controls of PGC-1α immunofluorescence were similar in both vehicle and melatonin cryopass-laser-treated mice; Figure 6D is shows the vehicle cryopass-laser-treated xenograft.

**Figure 7:**
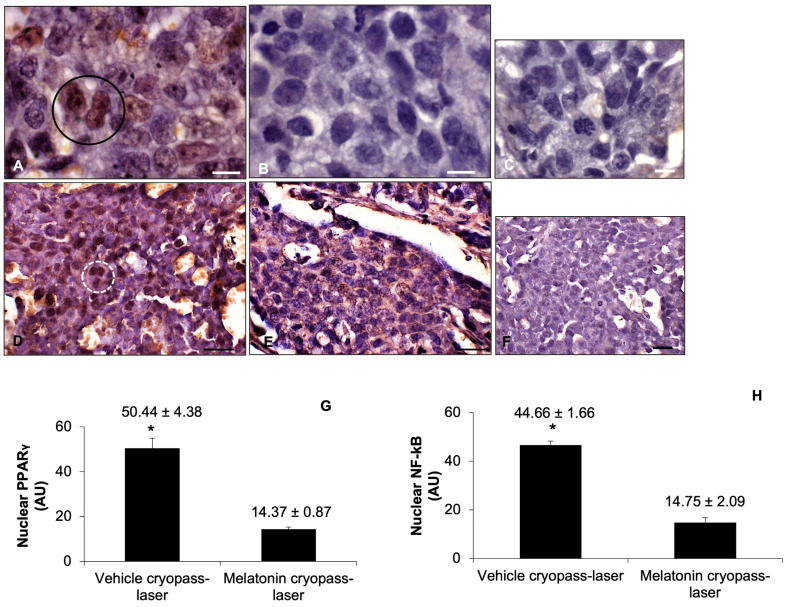
Representative photomicrographs of xenograft PPARγ (**A**–**C**) and NF-kB (**D**–**F**) immunostainings of vehicle cryopass-laser-treated mice (**A**,**D**) and melatonin cryopass-laser-treated mice (**B**,**E**). Representative photomicrographs of the negative controls of PPARγ (**C**) and of NF-kB (**F**) immunohistochemistry–vehicle cryopass-laser-treated group. Bars: 8 μm for PPARγ and 20 μm for NF-kB. The black circle represents PPARγ-positive tumor cell nuclei, whereas the white discontinuous circle indicates NF-kB-positive tumor cell nuclei. The graphs summarize PPARγ (**G**) and NF-kB (**H**) immunomorphometrical measurements, respectively. * *p* ≤ 0.05 vs. melatonin cryopass-laser group. PPARγ: peroxisome proliferator-activated receptor gamma; NF-kB: nuclear factor kappa B.

To evaluate the SIRT1-related pathway in tumorigenesis in depth, we investigated both the PPARγ and NF-kB expressions. In detail, we observed that vehicle cryopass-laser-treated xenograft presents a strong/moderate PPARγ expression (Figure 7A), whereas melatonin cryopass-laser treatment of xenografted mice exhibited a significant decrease in this protein, showing a very weak expression (Figure 7B). In particular, evaluating the different PPARγ positivity degrees among vehicle and melatonin cryopass-laser, we observed that this downstream protein is more expressed in the nuclei of vehicle-treated tumor cells, with a significant difference compared to melatonin-treated tumors. The negative control of PPARγ immunostaining is shown in Figure 7C. Furthermore, NF-kB positivity was strongly expressed in the tumor cell nuclei of vehicle cryopass-laser-treated xenografts (Figure 7D). Conversely, in melatonin cryopass-laser-treated xenografts, NF-kB immunopositivity was weak at the tumor cell’s nuclear level (Figure 7E). The negative controls of NF-kB immunohistochemistry were similar in both vehicle and melatonin cryopass-laser-treated mice; Figure 7F shows the vehicle cryopass-laser-treated xenograft.

The observations reported above were confirmed by the immunomorphometrical analysis of xenograft PPARγ and NF-kB expression (respectively Figure 7G,H). 

## 4. Discussion

The most innovative information from this study is that melatonin, a pineal product, administered by cryopass-laser, a procedure used to actively deliver drugs at the site of action, can reduce the growth of LNCaP tumor cells xenografted in nude mice by affecting SIRT1 expression, which can influence the PGC-1α/PPAR-γ pathway, NF-κB signaling, neovascularization, and proliferation.

Moreover, using the Sirius red and polarized light methods, we quantitatively validated the higher abundance of total collagen in melatonin cryopass-laser-treated tumors compared to controls. Further analysis of polarized light images confirms a higher abundance of type III collagen in melatonin-treated tumors.

Tumor microenvironment (TME), the intermediary between biomechanics and tumor biology, can have a dual role as a tumor suppressor in the early stages and as a tumor promoter in the later stages of tumor progression, likely due to ineffective T cell migration and penetration into the tumor mass, among other mechanisms [27,28]. The tumors seem to be at a very early stage and the mouse model used is lacking the ability to develop mature T-cells. Therefore, we can suppose a protective role of TME in these mice at this stage.

Our data agree with previous work that showed a decrease in type III collagen in the stromal microenvironment, which increased the aggressiveness of proliferative tumors, whereas upregulated type III collagen gene expression is associated with increased survival in patients with breast cancer, suggesting that type III collagen can limit metastasis [29,30]. Moreover, Ageeli et al. [31] also showed that in prostate cancer, there is a correlation between collagen type and content, including its orientation, and prostate cancer aggressivity. Differences in collagen content, orientation, distribution, structures, and type play a critical role in prostate cancer progression, demonstrating, by gene expression, that the amount of COL1A1 increased in cancer tissue compared to COL3A1. 

Furthermore, we can suppose that the increase in type collagen III content in cryopass-laser melatonin-treated tumors could be responsible for the lack of significance in tumor dimension reduction. 

Our results showed that prostate tumor xenografted overexpressed SIRT1. Contradictory information has been reported on the role of SIRT1 both in vivo and in vitro. In fact, SIRT1 is closely related to cell growth increase and proliferation inhibition [32,33,34,35,36,37], so this protein seems to have both tumorigenic and oncostatic effects, likely depending on the context [38,39,40].

Our results agree with the data of Huang et al. 2021 [40], who demonstrated that orthotopical implantation of SIRT1-silenced LNCaP cells in mice produced tumors that were reduced in size and weight and showed increased latency of development.

Moreover, SIRT1 directly interacts with and deacetylates PGC-1α, forming a transcriptional complex to determine the expression of other metabolic genes [41,42]. PGC1-α is a transcriptional co-activator that regulates the activity of transcription factors known to play a crucial role in mitochondrial function [43].

Tumor cells that strongly expressed PGC-1α are characterized by the ability to tackle reactive oxygen species (ROS) increase and toxicity; this, in turn, is beneficial to tumor cell proliferation and growth [44,45]. 

These data agree with our results that in prostate tumor xenografted tumors, there is also a high immunopositivity for PGC-1α.

Moreover, PGC-1α is the most well-studied regulator involved in tumor chemoresistance due to its action in tumor cell survival and metastasis under environmental stress [14,45,46,47,48]. 

PGC-1α is a transcriptional coactivator of the peroxisome proliferator-activated receptors (PPARs) superfamily, which also includes PPARγ. PGC-1α interacts with PPARγ and influences many other transcriptional factors that may affect health status [49]. 

In accordance with previously published data, we demonstrated that in xenograft tumors, there is also an increase in PPARγ protein expression. PPARγ was considered a tumor suppressor in prostate cells; conversely, new research has found that PPARγ antagonists inhibit cell growth. Furthermore, PPARγ expression increases with tumor grade/stage, suggesting that PPARγ activity may play a pro-tumorigenic role in prostate tumors [50]. Bao et al. [51] found that PPARγ inhibitor GW9662 reduced the growth, colony formation, and invasiveness of LNCaP cells. Another study in vitro by Ahmad et al. [52] has demonstrated that the knockdown of PPARγ in PC3-M cells by siRNA significantly reduced tumor size and incidence. In particular, suppression of PPARγ in prostate tumor cells reduces proliferation, invasiveness, and anchorage-independent growth. Furthermore, accumulated evidence has demonstrated that SIRT1-mediated deacetylation can activate PPARγ, suggesting a relation between SIRT1–PPARγ increase [14].

Moreover, our study demonstrated that melatonin could also modulate NF-κB signaling that is involved in cellular inflammation, and stress, as well as in the regulation of cell differentiation and proliferation. This was shown by a reduction in proliferation as demonstrated by the decrease in ki-67 and neovascularization.

NF-κB is the main switch of the inflammatory processes, which is usually connected to its inhibitory protein inhibitor of NF-κB (IκB) in the form of a p65/p50 heterodimer. When stimulated, the heterodimer can be activated and transferred to the nucleus, where it regulates the transcription of various downstream inflammatory factors. The p65 subunit of NF-κB is the direct target of SIRT1, which, through deacetylation, can regulate the transcription level of many inflammatory factors, thus regulating the inflammatory response [15].

So, melatonin administration via its SIRT1 inhibition could modulate and also reduce NF-kB expression.

Furthermore, according to our data, the melatonin-mediated reduction of SIRT1 could also affect the MMP2 expression in tumor cells, a zinc-dependent endopeptidase that has been shown to play an important role in extracellular matrix degradation, and thus in prostate tumor progression and tumor migration/invasion [16].

We can conclude that in the present study, we provided evidence for additional and synergic prostate tumor inhibitory activity of melatonin administered through the cryopass-laser route, confirming its pleiotropic potential. Inhibition of SIRT-1 and, in turn, modulation of collagen composition of ECM, PGC-1α/PPARγ pathway, and NF-kB signaling works in synergy with other cytoplasmic and nuclear activities [8,10]. 

Further studies should aim to evaluate if melatonin can improve prostate tumor prognosis in a direct manner or indirectly via the melatonin receptor 1 that was previously shown to be highly expressed on LNCaP cells’ surface [53].

Taken together, these results support further clinical studies on the therapeutic efficacy of cryopass-laser melatonin treatment in human tumors, and, in particular, on the therapeutic potential of inhibitors of SIRT1 toward prostate cancer. All the more, such inhibitors could be tested in this topical model or in vitro experiments with prostate cancer cell lines, also with the aim to assess the affect on SIRT1 posttranslational pathways. 

To date, pre-clinical studies and ongoing clinical trials are evaluating the potential effect of melatonin as an oncostatic molecule or in combination with currently approved therapies in different malignancies [54]. Moreover, cryopass-laser mediated delivery could constitute a promising safe and efficient alternative administration route [10,55]. 

Due to the anatomical proximity of the prostate to the anterior rectal wall, we can suppose that transrectal administration of melatonin using cryopass-laser could be an adjuvant therapy for prostate cancer. This whole-gland treatment could be an alternative or adjuvant therapy offering potential for oncological control with preservation of quality of life.

This study has some limitations: a) it would be interesting to evaluate the effect of cryopass-laser melatonin treatment at longer treatment times, as well as using more than one prostate cancer cell line xenografts; b) it would be interesting to confirm our findings with Western Blot or ELISA assays directly on the tumor tissue. Unfortunately, the samples used for these experiments were immediately fixed after the collection.

## 5. Conclusions

We have previously studied the relationship between the melatonin-reducing effect on tumor growth and hypoxia signaling in vivo, demonstrating that it is also effective against tumor progression at very low plasma levels [8]. 

The results of this study suggest that melatonin administered topically via cryopass-laser could decrease tumor growth in tumor-bearing mice in a complex manner, specifically affecting SIRT1 expression, which, in turn, modulates and orchestrates different pathways (i.e., PGC-1α/PPARγ pathway and NF-kB signaling) involved in tumor growth and progression. Moreover, this indoleamine has shown an effect on collagen content and MMP2 positivity in tumors, suggesting a broader effect on tumor microenvironment.

## Figures and Tables

**Table 1:** Body weight (BW) and tumor volume (TV) data.

		Melatonin Cryopass-Laser			Vehicle Cryopass-Laser		
	Days	BW (g)	TV (mm^3^)	TV/BW (mm^3^/g)	BW (g)	TV (mm^3^)	TV/BW (mm^3^/g)
Time after tumor cell inoculum	2	27.4± 0.5	0	0	27.5 ± 0.5	0	0
	12	29.5± 0.7	0	0	29.2 ± 0.8	0	0
	22	29.3 ± 0.9	45 ± 16	1.62 ± 0.6	29.0 ± 1.2	49 ± 21	1.81 ± 0.83
	32	28.3 ± 0.9	85 ± 19	3.14 ± 0.7	27.7 ± 1.3	89 ± 37	3.53 ± 1.5
	42	28.3 ± 1	131 ± 34	4.82 ± 1.3	27.5 ± 1.4	151 ± 47	5.94 ± 1.9

## Data Availability

The data underlying this article will be shared upon reasonable request to the corresponding author.

## References

[1] World Health Organization Cancer. World Health Organization: Geneva, Switzerland.

[2] Leslie S.W., Soon-Sutton T.L., Sajjad H., Siref L.E. (2022). Prostate Cancer. StatPearls.

[3] Kaiser A., Haskins C., Siddiqui M.M., Hussain A., D’Adamo C. (2019). The evolving role of diet in prostate cancer risk and progression. Curr. Opin. Oncol..

[4] Jha G.G., Anand V., Soubra A., Konety B.R. (2014). Challenges of managing elderly men with prostate cancer. Nat. Rev. Clin. Oncol..

[5] Long M.D., Singh P.K., Russell J.R., Llimos G., Rosario S., Rizvi A., van den Berg P.R., Kirk J., Sucheston-Campbell L.E., Smiraglia D.J. (2019). The miR-96 and RARγ signaling axis governs androgen signaling and prostate cancer progression. Oncogene.

[6] Cattley R.C., Radinsky R.R. (2004). Cancer therapeutics: Understanding the mechanism of action. Toxicol. Pathol..

[7] Davoodvandi A., Nikfar B., Reiter R.J., Asemi Z. (2022). Melatonin and cancer suppression: Insights into its effects on DNA methylation. Cell. Mol. Biol. Lett..

[8] Paroni R., Terraneo L., Bonomini F., Finati E., Virgili E., Bianciardi P., Favero G., Fraschini F., Reiter R.J., Rezzani R. (2014). Antitumour activity of melatonin in a mouse model of human prostate cancer: Relationship with hypoxia signalling. J. Pineal Res..

[9] Karasek M. (2007). Does melatonin play a role in aging processes?. J. Physiol. Pharmacol..

[10] Terraneo L., Bianciardi P., Virgili E., Finati E., Samaja M., Paroni R. (2017). Transdermal administration of melatonin coupled to cryopass laser treatment as noninvasive therapy for prostate cancer. Drug Deliv..

[11] Haigis M.C., Guarente L.P. (2006). Mammalian sirtuins: Emerging roles in physiology, aging, and calorie restriction. Genes. Dev..

[12] Shi Y., Han J.J., Tennakoon J.B., Mehta F.F., Merchant F.A., Burns A.R., Howe M.K., McDonnell D.P., Frigo D.E. (2013). Androgens promote prostate cancer cell growth through induction of autophagy. Mol. Endocrinol..

[13] Pulla V.K., Alvala M., Sriram D.S., Viswanadha S., Sriram D., Yogeeswari P. (2014). Structure-based drug design of small molecule SIRT1 modulators to treat cancer and metabolic disorders. J. Mol. Graph. Model..

[14] Xu R., Luo X., Ye X., Li H., Liu H., Du Q., Zhai Q. (2021). SIRT1/PGC-1α/PPAR-γ correlate with hypoxia-induced chemoresistance in non-small cell lung cancer. Front. Oncol..

[15] Ren Z., He H., Zuo Z., Xu Z., Wei Z., Deng J. (2019). The role of different SIRT1-mediated signaling pathways in toxic injury. Cell. Mol. Biol. Lett..

[16] Onyiba C.I., Scarlett C.J., Weidenhofer J. (2022). The Mechanistic Roles of Sirtuins in Breast and Prostate Cancer. Cancers.

[17] Burns-Cox N., Avery N.C., Gingell J.C., Bailey A.J. (2001). Changes in collagen metabolism in prostate cancer: A host response that may alter progression. J. Urol..

[18] Szadvari I., Krizanova O., Babula P. (2016). Athymic nude mice as an experimental model for cancer treatment. Physiol. Res..

[19] Favero G., Lonati C., Giugno L., Castrezzati S., Rodella L.F., Rezzani R. (2013). Obesity-related dysfunction of the aorta and prevention by melatonin treatment in ob/ob mice. Acta Histochem..

[20] Favero G., Paini A., De Ciuceis C., Rodella L.F., Moretti E., Porteri E., Rossini C., Ministrini S., Solaini L., Stefano C. (2018). Changes in extracellular matrix in subcutaneous small resistance arteries of patients with essential hypertension. Blood Press..

[21] Bonomini F., Favero G., Rodella L.F., Moghadasian M.H., Rezzani R. (2018). Melatonin Modulation of Sirtuin-1 Attenuates Liver Injury in a Hypercholesterolemic Mouse Model. BioMed Res. Int..

[22] Stacchiotti A., Favero G., Giugno L., Golic I., Korac A., Rezzani R. (2017). Melatonin Efficacy in Obese Leptin-Deficient Mice Heart. Nutrients.

[23] Favero G., Franco C., Stacchiotti A., Rodella L.F., Rezzani R. (2020). Sirtuin1 Role in the Melatonin Protective Effects Against Obesity-Related Heart Injury. Front. Physiol..

[24] Rodella L.F., Rossini C., Favero G., Foglio E., Loreto C., Rezzani R. (2012). Nicotine-induced morphological changes in rat aorta: The protective role of melatonin. Cells Tissues Organs.

[25] Watanabe R., Maekawa M., Kiyoi T., Kurata M., Miura N., Kikugawa T., Higashiyama S., Saika T. (2021). PSMA-positive membranes secreted from prostate cancer cells have potency to transform vascular endothelial cells into an angiogenic state. Prostate.

[26] Inoue R., Yasuma T., Fridman D’Alessandro V., Toda M., Ito T., Tomaru A., D’Alessandro-Gabazza C.N., Tsuruga T., Okano T., Takeshita A. (2023). Amelioration of Pulmonary Fibrosis by Matrix Metalloproteinase-2 Overexpression. Int. J. Mol. Sci..

[27] Nicolas-Boluda A., Vaquero J., Vimeux L., Guilbert T., Barrin S., Kantari-Mimoun C., Ponzo M., Renault G., Deptula P., Pogoda K. (2021). Tumor stiffening reversion through collagen crosslinking inhibition improves T cell migration and anti-PD-1 treatment. Elife.

[28] Torzilli P.A., Bourne J.W., Cigler T., Vincent C.T. (2012). A new paradigm for mechanobiological mechanisms in tumor metastasis. Semin. Cancer Biol..

[29] Beck A.H., Espinosa I., Gilks C.B., van de Rijn M., West R.B. (2008). The fibromatosis signature defines a robust stromal response in breast carcinoma. Lab. Investig..

[30] Di Martino J.S., Nobre A.R., Mondal C., Taha I., Farias E.F., Fertig E.J., Naba A., Aguirre-Ghiso J.A., Bravo-Cordero J.J. (2022). A tumor-derived type III collagen-rich ECM niche regulates tumor cell dormancy. Nat. Cancer.

[31] Ageeli W., Zhang X., Ogbonnaya C.N., Ling Y., Wilson J., Li C., Nabi G. (2021). Characterisation of Collagen Re-Modelling in Localised Prostate Cancer Using Second-Generation Harmonic Imaging and Transrectal Ultrasound Shear Wave Elastography. Cancers.

[32] Kojima K., Ohhashi R., Fujita Y., Hamada N., Akao Y., Nozawa Y., Deguchi T., Ito M. (2008). A role for SIRT1 in cell growth and chemoresistance in prostate cancer PC3 and DU145 cells. Biochem. Biophys. Res. Commun..

[33] Jung-Hynes B., Nihal M., Zhong W., Ahmad N. (2009). Role of sirtuin histone deacetylase SIRT1 in prostate cancer. A target for prostate cancer management via its inhibition?. J. Biol. Chem..

[34] Ruan L., Wang L., Wang X., He M., Yao X. (2018). SIRT1 contributes to neuroendocrine differentiation of prostate cancer. Oncotarget.

[35] Byles V., Zhu L., Lovaas J.D., Chmilewski L.K., Wang J., Faller D.V., Dai Y. (2012). SIRT1 induces EMT by cooperating with EMT transcription factors and enhances prostate cancer cell migration and metastasis. Oncogene.

[36] Wang X., Yang B., Ma B. (2016). The UCA1/miR-204/Sirt1 axis modulates docetaxel sensitivity of prostate cancer cells. Cancer Chemother. Pharmacol..

[37] Li G., Rivas P., Bedolla R., Thapa D., Reddick R.L., Ghosh R., Kumar A.P. (2013). Dietary resveratrol prevents development of high-grade prostatic intraepithelial neoplasticlesions: Involvement of SIRT1/S6K axis. Cancer Prev. Res..

[38] Chalkiadaki A., Guarente L. (2015). The multifaceted functions of sirtuins in cancer. Nat. Rev. Canc..

[39] Wilking M.J., Ahmad N. (2015). The role of SIRT1 in cancer: The saga continues. Am. J. Pathol..

[40] Huang S.B., Thapa D., Munoz A.R., Hussain S.S., Yang X., Bedolla R.G., Osmulski P., Gaczynska M.E., Lai Z., Chiu Y.C. (2021). Androgen deprivation-induced elevated nuclear SIRT1 promotes prostate tumor cell survival by reactivation of AR signaling. Cancer Lett..

[41] Zhu H.R., Wang Z.Y., Zhu X.L., Wu X.X., Li E.G., Xu Y. (2010). Icariin protects against brain injury by enhancing SIRT1-dependent PGC-1alpha expression in experimental stroke. Neuropharmacology.

[42] Zhang Q., Chen W., Xie C., Dai X., Ma J., Lu J. (2020). The Role of PGC-1α in Digestive System Malignant Tumours. Anti-Cancer Agents Med. Chem..

[43] Wu N., Jin W., Zhao Y., Wang H., He S., Zhang W., Zhou J. (2022). Sulfated Fucogalactan from Laminaria Japonica Ameliorates β-Cell Failure by Attenuating Mitochondrial Dysfunction via SIRT1-PGC1-α Signaling Pathway Activation. Front. Endocrinol..

[44] Luo C., Widlund H.R., Puigserver P. (2016). PGC-1 Coactivators: Shepherding the Mitochondrial Biogenesis of Tumors. Trends Cancer.

[45] Zheng K., Chen S., Hu X. (2022). Peroxisome Proliferator-activated Receptor Gamma Coactivator-1 Alpha: A Double-edged Sword in Prostate Cancer. Curr. Cancer Drug Targets.

[46] Yun C.W., Han Y.-S., Lee S.H. (2019). PGC-1α Controls mitochondrial biogenesis in drug-resistant colorectal cancer cells by regulating endoplasmic reticulum stress. Int. J. Mol. Sci..

[47] Jiang J., Wang K., Chen Y., Chen H., Nice E.C., Huang C. (2017). Redox regulation in tumor cell epithelial-mesenchymal transition: Molecular basis and therapeutic strategy. Signal Transduct. Target. Ther..

[48] Kim B., Jung J.W., Jung J., Han Y., Suh D.H., Kim H.S., Dhanasekaran D.N., Song Y.S. (2017). PGC1alpha induced by reactive oxygen species contributes to chemoresistance of ovarian cancer cells. Oncotarget.

[49] Montes-de-Oca-García A., Corral-Pérez J., Velázquez-Díaz D., Perez-Bey A., Rebollo-Ramos M., Marín-Galindo A., Gómez-Gallego F., Calderon-Dominguez M., Casals C., Ponce-González J.G. (2022). Influence of Peroxisome Proliferator-Activated Receptor (PPAR)-gamma Coactivator (PGC)-1 alpha gene rs8192678 polymorphism by gender on different health-related parameters in healthy young adults. Front. Physiol..

[50] Elix C., Pal S.K., Jones J.O. (2018). The role of peroxisome proliferator-activated receptor gamma in prostate cancer. Asian J. Androl..

[51] Bao Z., Malki M.I., Forootan S.S., Adamson J., Forootan F.S., Chen D., Foster C.S., Rudland P.S., Ke Y. (2013). A novel cutaneous fatty acid-binding protein-related signaling pathway leading to malignant progression in prostate cancer cells. Genes Cancer.

[52] Ahmad I., Mui E., Galbraith L., Patel R., Tan E.H., Salji M., Rust A.G., Repiscak P., Hedley A., Markert E. (2016). Sleeping Beauty screen reveals Pparg activation in metastatic prostate cancer. Proc. Natl. Acad. Sci. USA.

[53] Xi S.C., Siu S.W., Fong S.W., Shiu S.Y. (2001). Inhibition of androgen-sensitive LNCaP prostate cancer growth in vivo by melatonin: Association of antiproliferative action of the pineal hormone with mt1 receptor protein expression. Prostate..

[54] Wang L., Wang C., Choi W.S. (2022). Use of Melatonin in Cancer Treatment: Where Are We?. Int. J. Mol. Sci..

[55] Biava P.M., Bonizzoni E., Zafiropoulou S., Laudani A., Burigana F., Burian Lissoi I., Lotti T. (2020). Stem cell growth and differentiation factors from Zebrafish embryo and their role as epigenetic regulators in hair regeneration: Results after transdermal administration using cryopass laser treatment. Aesthetic Med..

